# Giant Mesenchymal Hepatic Hamartomas With Adrenal Involvement Precipitating Respiratory Failure: A Myxomatous Mystery in a Three-Month-Old

**DOI:** 10.7759/cureus.37476

**Published:** 2023-04-12

**Authors:** Ali Yasback, Abid Ulhaque, Tushar Chandra

**Affiliations:** 1 College of Allopathic Medicine, Nova Southeastern University Dr. Kiran C. Patel College of Allopathic Medicine, Fort Lauderdale, USA; 2 Radiology, HCA West Florida, Trinity Medical Center, Trinity, USA; 3 Pediatric Radiology, Nemours Children's Hospital, Orlando, USA

**Keywords:** cysts, placental mesenchymal dysplasia, oncology, pediatric tumors, hepatic mesenchymal hamartoma

## Abstract

The combination of placental mesenchymal dysplasia and hepatic mesenchymal hamartomas is an extremely rare finding. We present the case of a three-month-old female born at 35 weeks gestation with a history of placental mesenchymal dysplasia who presented with non-bilious, non-bloody emesis, and episodes of respiratory distress due to multiple enlarging abdominal cystic lesions. The patient’s presentation was unique due to both liver and adrenal solid and cystic lesions. After extensive imaging and multiple biopsies, expert interpretation of biopsy tissue revealed hepatic mesenchymal hamartoma within the liver and the adrenal gland. To our knowledge, this is one of the few documented cases of unresectable hepatic mesenchymal hamartomas with adrenal involvement successfully undergoing a whole liver transplant.

## Introduction

Hamartomas are benign, local proliferations of disorganized tissue. Hepatic mesenchymal hamartomas (HMHs) are therefore a proliferation of mesenchymal liver tissue. They are not often associated with elevated alpha-fetoprotein (AFP) levels though there are case reports of HMH noting elevated AFP levels [1]. Although hamartomas are benign, they can ultimately lead to fetal demise both in utero and perinatally [2,3]. This is often due to respiratory failure secondary to mass effect on the diaphragm and lungs or compression of other vital organs [4]. Rarely, HMH may present with placental mesenchymal dysplasia (PMD). HMHs have been described to be intrahepatic, exophytic, and rarely extra-hepatic [4].

## Case presentation

We present the case of a three-month-old girl born at 35 weeks with known hepatic masses/cysts and an adrenal cyst, brought to the emergency department by her mother due to four days of nonbilious, non-bloody emesis. The patient was undergoing a workup for hepatic lesions at an outside hospital. Prenatal sonograms showed maternal placental mesenchymal dysplasia and multiple abdominal cysts within the patient’s liver. The patient was delivered via cesarean section due to nonreassuring fetal heart rates, and the pregnancy was otherwise uncomplicated. Following the delivery, the patient’s liver cysts were followed closely. Interval sonograms revealed enlarging complex cysts and an enlarging adrenal lesion was also discovered. The patient was discharged with a follow-up ultrasound scheduled.

**Figure 1:**
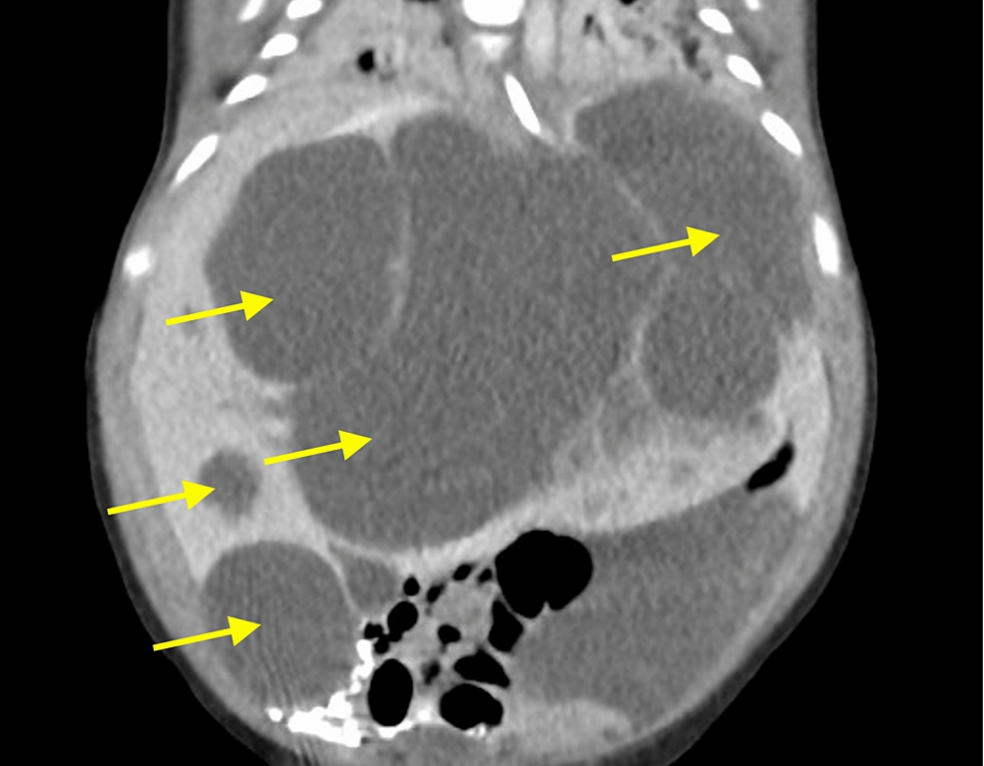
Coronal enhanced CT demonstrates multiple non-enhancing fluid attenuating cysts in the liver causing mass effect

**Figure 2:**
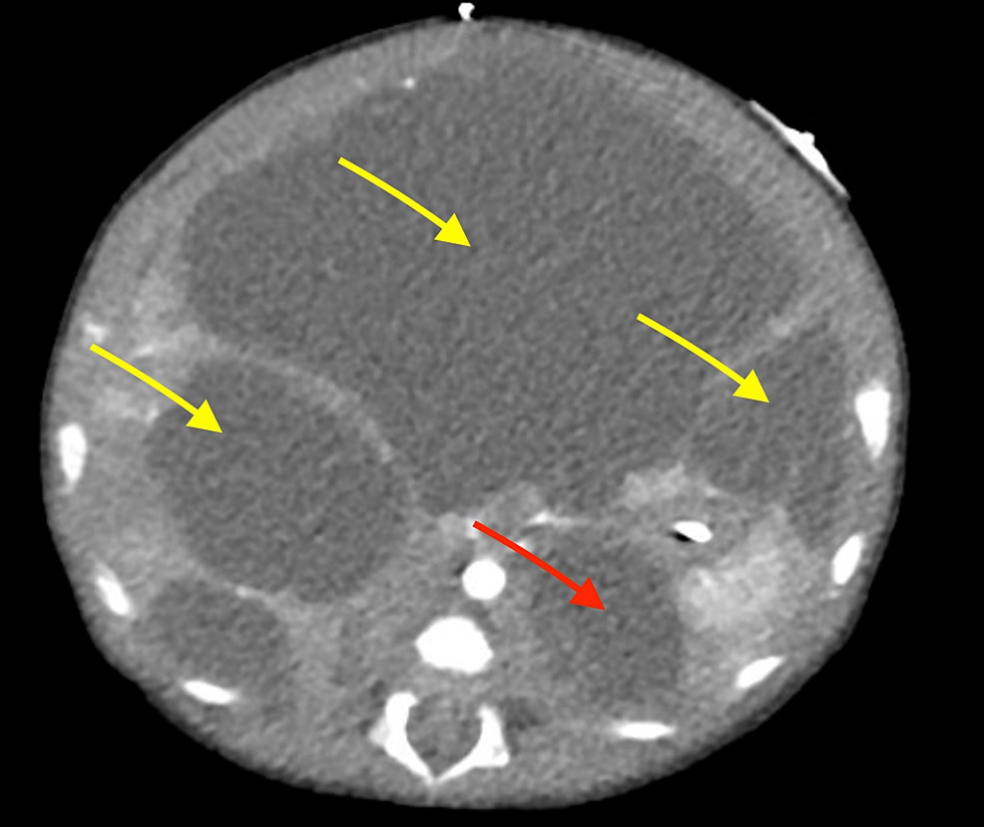
Axial enhanced CT demonstrates large, well-circumscribed, fluid-attenuating cysts in the liver causing mass effect (yellow) There is a cyst seen in the left para-renal/retroperitoneal region (red).

**Figure 3:**
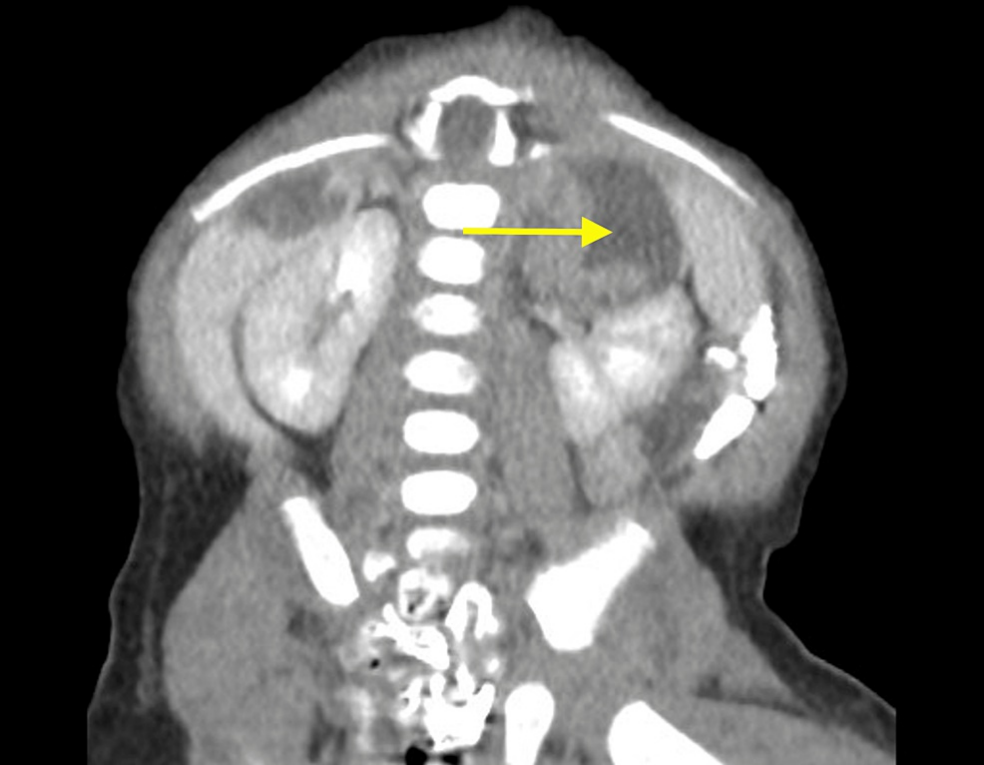
Coronal enhanced CT demonstrates well-circumscribed, fluid-attenuating cysts lateral/posterior margin of the liver There is a cyst seen in the left para-renal region, which appears to be contiguous with the left adrenal gland.

Over the next two months, the patient frequently returned to the hospital and was admitted multiple times for an inability to feed and tachypnea. Extensive imaging, including CT scans, was performed to monitor the growth of the lesions (Figures 1-3). A nasogastric tube was used to guide feeds, and supplemental oxygen was given as needed. At an outside hospital, laparoscopic aspiration of the biggest liver cyst was performed to decrease the mass effect. Biopsies of the liver and left adrenal lesion were also performed. Pathology was nondiagnostic with extensive fibromyxoid component, making neuroblastoma an unlikely diagnosis. An iodine-123 metaiodobenzylguanidine (MIBG) scan, which is used to identify neuroendocrine tumors, was negative, making a neuroendocrine tumor unlikely. Other adrenal or hamartomatous neoplasms/lesions could not be excluded. Pathology samples were sent to a large, out-of-state academic children’s center for a second opinion and bone marrow aspirate preliminarily showed normal trilinear maturation without evidence of metastatic tumor.

**Figure 4:**
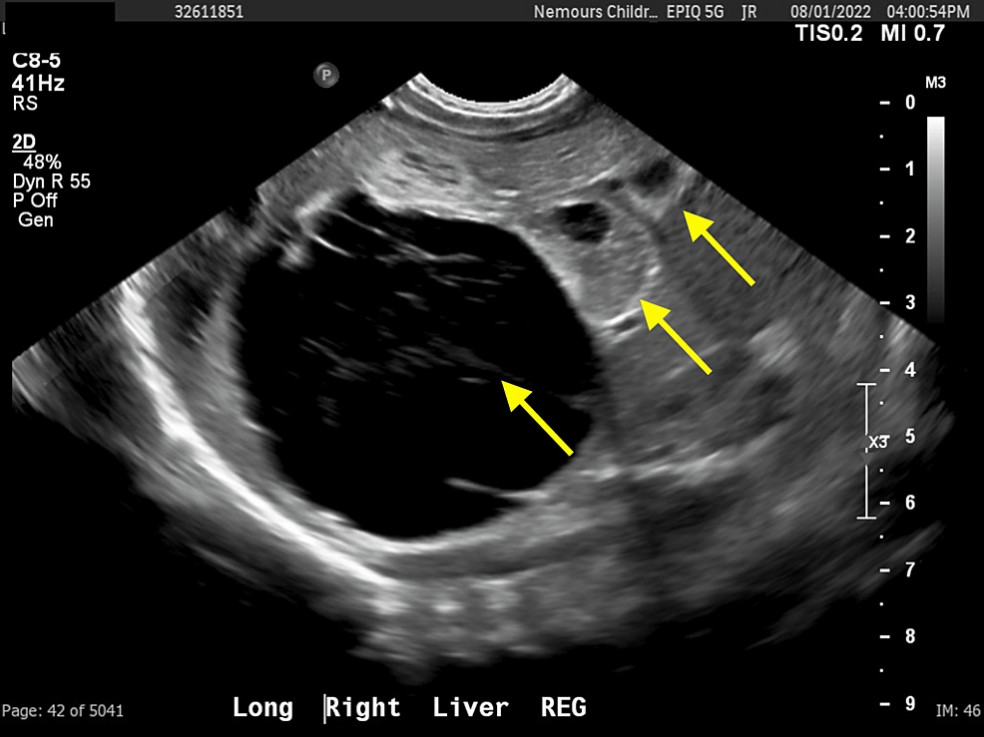
Sonographic image of the patient’s liver demonstrates a well-circumscribed, multiseptated, anechoic cyst surrounded by smaller mixed solid and cystic lesions

**Figure 5:**
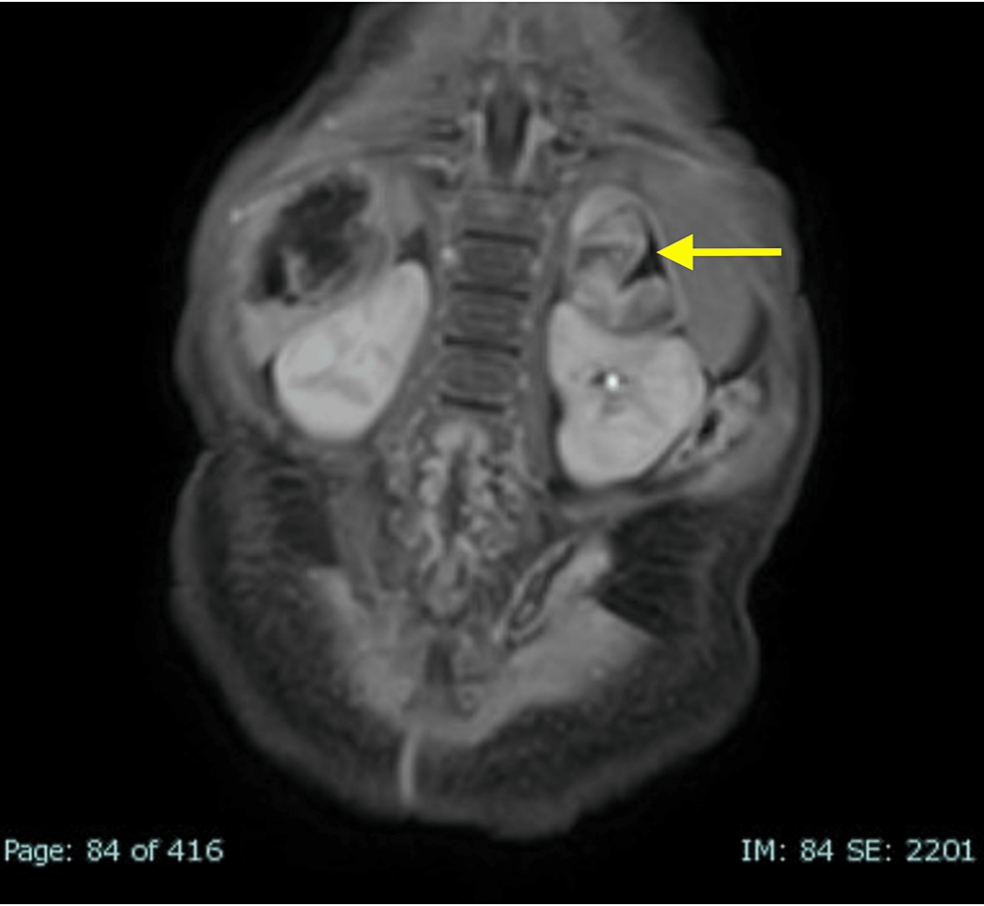
Contrast-enhanced T1 coronal view demonstrates a left heterogeneous adrenal lesion that is seen distinct from the left kidney (no claw-sign) The lesion has an enhancement of solid and a non-enhancement of cystic portions.

**Figure 6:**
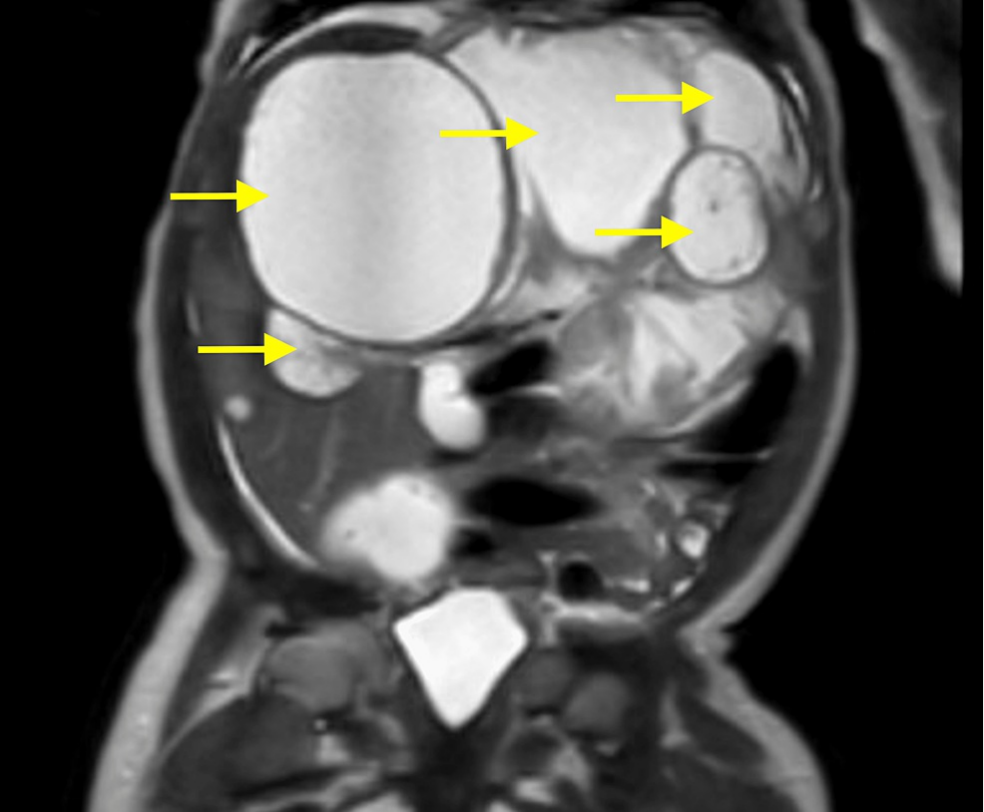
T2 coronal view shows multiple fluid-filled cysts in the liver causing mass effect

The patient’s parents decided to relocate to be closer to family and an appointment with the oncologist resulted in an ultrasound and magnetic resonance imaging (MRI) evaluation. The ultrasound showed multiple, complex, predominantly cystic lesions within the abdomen, retroperitoneum, liver (Figure 4), and left adrenal gland. Further evaluation by MIBG was recommended and an 18F-FDG PET/CT (fluorine-18 fluorodeoxyglucose positron emission tomography/computed tomography) was suggested as cystic neuroblastoma can be MIBG negative. MRI abdomen showed peritoneal, left adrenal, and multiple hepatic cystic lesions with the largest cyst measuring 11.2 by 8.1 cm (Figures 5-6). During the workup, an alpha-fetoprotein (AFP) was elevated to 5760 ng/mL with a local reference range of (0-339). Elevated levels of AFP are typically indicative of malignancy such as neuroblastoma, and this further complicates diagnosis. Throughout her hospital stay, which lasted over a month, the patient remained persistently microcytically anemic. Human chorionic gonadotropic (HCG), carcinoembryonic antigen (CEA), Coombs test, gamma-glutamyl transferase (GGT), adrenocorticotropic hormone (ACTH), urine vanillylmandelic acid (VMA), homovanillic acid (HVA), and random cortisol were within normal limits.

Following the MRI, an ultrasound-guided biopsy of the left adrenal lesion was performed with a total of 10 core samples. The hospital pathologist interpreted the samples as myxoid adrenal cortical tumors with cellular architecture resembling adenoid cystic carcinoma. The cells were positive for MART1, CD56, synaptophysin, and inhibin. A focal positivity of calretinin was noted. Chromogranin, AFP, cytokeratin, MSA, CD45, and CD31 were negative. The cells were seen in a mucinous background and strongly positive for Alcian blue, but negative for S-100. Following this diagnosis, the oncology team requested a re-evaluation of the patient’s pathology reports, and tissue samples were subsequently sent to pathologists around the country for second opinions. Expert analysis from St. Judes returned a new diagnosis of mesenchymal hamartoma. The oncologic workup continued with the Tempus molecular testing assay (Chicago, IL) being negative (including testing for Beckwith-Wiedemann syndrome). The patient was then transferred to a sister facility out of state for liver transplantation evaluation, as her cysts were enlarging and affecting her ability to breathe. At the time of this writing, the patient received a cadaveric liver transplant with whole liver and duct-to-duct anastomosis. She experienced mild T-cell-mediated rejection successfully treated with steroids and is now gaining weight appropriately without other complications.

## Discussion

Hepatic mesenchymal hamartomas (HMHs) are benign tumors classically characterized by primitive myxoid stroma with associated dilated bile ducts. It is currently believed that these lesions stem from an alteration of the 19q13 chromosome. These lesions have been associated with both Beckwith-Wiedemann syndrome and placental mesenchymal dysplasia in the literature [5]. Most commonly, these lesions present as painless enlarging hepatic cystic lesions [6]. On ultrasound, HMHs present as both solid and cystic masses of varying sizes. Later in its presentation, the cystic lesions predominate in size and can be both uni- and multicystic. The solid components are seen adjacent to the cysts, as noted in our patient. The thickness of the septa is variable, and the color Doppler most commonly shows low blood flow or absent flow. High blood flow has previously been noted in the literature as a source of misdiagnosis in favor of hemangioma [7]. MRI is used for further evaluation of the lesions due to its high resolution of the soft tissues.

There are nine reported cases in the literature of combined placental mesenchymal dysplasia (PMD) and HMH and the combination of the two conditions is associated with a poorer prognosis of the fetus [2]. Due to the rarity of these conditions, especially when in conjunction, there is no established preferred treatment plan. Conservative treatment, surgical resections, and percutaneous drainage have all been employed in management. Of the nine reported cases, four of the patients survived with three of them undergoing surgical resection and one percutaneous cyst drainage [2,3,8-11]. There is currently no case report of combined PMD and HMH surviving without intervention or undergoing liver transplantation. In most cases, complete resection of the lesions is attempted. For unresectable lesions, a liver transplant is the treatment of choice [4].

HMH can be a challenging diagnosis for several reasons, including its unclear etiology, varying levels of AFP, and in this case, adrenal involvement with an ambiguous first biopsy. The literature describes a report of HMH located exophytically and extending inferiorly into the pelvis, which may be confused with an ovarian cystic lesion [4]. There have been cases described showing high flow to the cystic lesions, leading to misdiagnosis of hemangiomas and lymphangiomas [4]. In our patient, the left adrenal lesion became a distractor. The most common adrenal lesion that can metastasize is a neuroblastoma. Although our patient's AFP was elevated, an MIBG scan was negative, effectively ruling out this differential. The in-house pathology evaluation of the adrenal lesions was inconclusive, initially interpreting them as myxoid adrenal cortical tumors. Relevant staining was otherwise negative. Upon evaluation by a nationally renowned outside facility, the interpretation was consistent with HMH. The report described the following: “Although the fibromyxoid tissue in the liver had similar histological appearance as that in the adrenal gland, immunostaining of the liver did not show any tissue of adrenocortical origin.” It is thought that the patient has an adrenal origin of a hepatic mesenchymal hamartoma. To our knowledge, this has only sparsely been described in the literature and may suggest a malignant potential in an otherwise benign lesion. Furthermore, this is one of only a few cases in the literature of a patient successfully undergoing a whole liver transplant for HMH as opposed to surgical resection or percutaneous drainage [12,13].

## Conclusions

Hepatic mesenchymal hamartomas (HMHs) are the second most common benign hepatic tumors after hemangiomas. They are rare in occurrence and while they have been described in the literature, due to their atypical presentation, they can create diagnostic challenges. As seen in this case, an adrenal cystic lesion presented a distractor for the clinicians. Ultimately, expert interpretation of the biopsy showed the adrenal involvement of HMH.
